# Red Cell Distribution Width as a Predictive Biomarker for Early Lung Injury in Pediatric Patients Following Cardiopulmonary Bypass

**DOI:** 10.3390/children12060785

**Published:** 2025-06-16

**Authors:** Hui Liu, Jie Cheng, Kaicheng Peng, Lin Chen, Zhenxuan Kong, Yan Zhao, Zhengxiu Luo

**Affiliations:** 1 Department of Respiratory Medicine, Children’s Hospital of Chongqing Medical University, National Clinical Research Center for Child Health and Disorders, Ministry of Education Key Laboratory of Child Development and Disorders, Chongqing Key Laboratory of Child Rare Diseases in Infection and Immunity, Chongqing 400014, China; 2023130278@stu.cqmu.edu.cn (H.L.); pengkaicheng55@163.com (K.P.); cl18223456@163.com (L.C.); 2022110405@stu.cqmu.edu.cn (Z.K.); zhaoyanwow@126.com (Y.Z.); 2 Emergency Department, Children’s Hospital of Chongqing Medical University, National Clinical Research Center for Child Health and Disorders, Ministry of Education Key Laboratory of Child Development and Disorders, Chongqing Key Laboratory of Child Rare Diseases in Infection and Immunity, Chongqing 400014, China; 485159@hospital.cqmu.edu.cn

**Keywords:** acute lung injury (ALI), red cell distribution width (RDW), cardiopulmonary bypass (CPB), pediatric cardiac surgery, biomarker

## Abstract

**Background:** Red cell distribution width (RDW) has emerged as a prognostic biomarker in various clinical contexts. This retrospective study evaluated the predictive utility of RDW for cardiopulmonary bypass-associated acute lung injury (CPB-ALI) in pediatric patients undergoing cardiac surgery. **Methods:** A total of 166 children were enrolled and classified into CPB-ALI and non-ALI groups. Preoperative and postoperative RDW values were analyzed. **Results:** Postoperative RDW was significantly higher in the CPB-ALI group (15.40% vs. 13.78%, *p* < 0.001). Multivariate logistic regression identified postoperative RDW as an independent predictor of CPB-ALI (OR: 1.35, 95% CI: 1.10–1.64, *p* = 0.003). Receiver operating characteristic analyses yielded an AUC of 0.732, and restricted cubic spline analyses revealed a nonlinear association between RDW and CPB-ALI risks (*p* < 0.001). Higher postoperative RDW levels were positively correlated with prolonged mechanical ventilation duration, ICU stay, and total hospital stay (*p* < 0.001 for all). **Conclusions:** These findings suggest that postoperative RDW is a cost-effective and accessible biomarker for the early identification of CPB-ALI and may inform individualized perioperative management in pediatric cardiac surgery.

## 1. Introduction

Congenital heart disease (CHD), affecting 8–12 per 1000 live births globally, remains one of the most common congenital anomalies in pediatric populations [1,2,3]. While advancements in cardiac surgery and perioperative care have significantly improved survival rates, postoperative complications such as cardiopulmonary bypass-associated acute lung injury (CPB-ALI) continue to impose substantial morbidity and mortality burdens [4,5,6,7]. CPB-ALI occurs in 12–50% of pediatric patients undergoing cardiac surgery, with up to 20% requiring prolonged mechanical ventilation (>48 h), leading to prolonged hospital stays, increased healthcare costs, and heightened mortality risks [8,9,10].

The etiology of CPB-ALI is multifactorial, involving ischemia–reperfusion injury from CPB, ventilator-induced lung injury, systemic inflammatory responses, and oxidative stress triggered by surgical trauma [11,12,13,14,15,16]. These processes disrupt alveolar–capillary integrity, induce interstitial edema, and promote inflammatory cell infiltration, culminating in pulmonary dysfunction [13,17]. Despite its clinical significance, the early prediction of CPB-ALI remains challenging due to the lack of reliable biomarkers, hindering timely interventions to mitigate adverse outcomes.

The red cell distribution width (RDW), a routinely measured hematologic parameter reflecting erythrocyte volume heterogeneity, is traditionally utilized for anemia classification [18]. Emerging research demonstrates a significant correlation between RDW levels and disease prognosis. An elevated RDW is associated with adverse outcomes in conditions such as acute myocardial infarction, heart failure, and coronary artery disease [19,20,21,22]. Furthermore, RDW has demonstrated predictive relevance in infectious diseases, liver disorders, and acute kidney injury [20,23,24]. In patients with acute respiratory distress syndrome (ARDS), high RDW levels have been independently linked to increased mortality and poorer clinical outcomes [25,26]. Additionally, an elevated RDW serves as an independent risk marker for lung injury in acute pancreatitis [27]. Notably, existing studies predominantly focus on RDW’s association with the infectious etiologies of lung injury, leaving its role in sterile inflammatory processes—such as CPB-ALI—largely unexplored.

Although RDW’s prognostic utility in adult cardiovascular and critical care settings is well-documented, its applicability to pediatric CPB-ALI remains undefined. Children exhibit distinct physiological and pathological responses to surgical stress and inflammation, necessitating tailored biomarker investigations. The purpose of this retrospective study is to evaluate postoperative RDW dynamics in pediatric patients undergoing CPB-assisted cardiac surgery and determine its predictive efficacy for CPB-ALI occurrence. By addressing this knowledge, we seek to establish RDW as a cost-effective, accessible biomarker for early risk stratification, thereby guiding personalized clinical management and improving postoperative outcomes in this vulnerable population.

## 2. Materials and Methods

### 2.1. Study Design and Population

**Figure 1:**
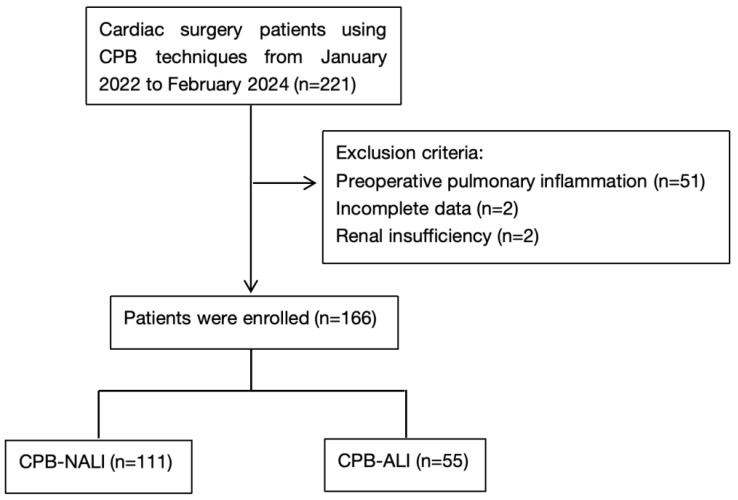
Flow chart of the study population. CPB-ALI, cardiopulmonary bypass–associated acute lung injury; CPB-NALI, no acute lung injury.

This single-center retrospective cohort study was conducted at the Children’s Hospital of Chongqing Medical University. The study’s protocol was approved by the hospital’s Ethics Committee (Approval No. [2025] Lunshen (Linyan) No. 88, approved on 20 March 2025) and conducted in accordance with the Declaration of Helsinki. The requirement for informed consent was waived due to the retrospective design. Patients aged 0–16 years who underwent cardiac surgery requiring cardiopulmonary bypass (CPB) between January 2022 and February 2024 were eligible for inclusion. Exclusion criteria comprised the following: (1) incomplete clinical or laboratory data; (2) preterm birth (gestational age <36 weeks); (3) preexisting liver or renal dysfunction; (4) severe chromosomal abnormalities; (5) preoperative pulmonary inflammation, pulmonary edema secondary to heart failure, or postoperative extracorporeal membrane oxygenation (ECMO) support; and (6) preoperative mechanical ventilation. After applying these criteria, 166 pediatric patients were included in the final analysis (Figure 1).

### 2.2. Data Collection and Variables

Demographic, clinical, and perioperative data were extracted retrospectively from electronic medical records and institutional databases. The collected variables included the following: demographic data (age, gender, and weight), clinical history (diagnosis and comorbidities), medical orders (long-term and temporary), laboratory data (RDW and arterial blood gases), imaging data (chest X-ray and chest CT), and perioperative data (surgery type, duration of CPB, aortic cross-clamp time, and duration of surgery). ICU length of stay (ICU LOS), hospital length of stay (hospital LOS), and postoperative complications were all comprehensively reviewed and analyzed. This approach allowed for a detailed examination of the potential predictors and outcomes associated with pediatric cardiac surgeries with CPB. Peripheral blood samples were analyzed using standard laboratory procedures at the central laboratory of the Children’s Hospital of Chongqing Medical University. Complete blood count parameters, including RDW, were measured using automated hematology analyzers (Abbott CD-1700, Medonic-620). The laboratory is equipped with internationally certified instruments and operates under strict quality control protocols.

### 2.3. Main Definitions

Based on the American-European Consensus Conference (AECC) definitions, ALI is defined as follows [28]:

• Oxygenation: PaO_2_/FiO_2_ < 300 mmHg (regardless of the level of positive end-expiratory pressure, PEEP);
• Chest radiograph: Presence of bilateral infiltrates;
• Pulmonary artery occlusion pressure (PAOP): Less than 18 mmHg, indicating no cardiogenic pulmonary edema (CPE).

Routine blood sampling and imaging studies, including chest X-rays or CT scans, were conducted preoperatively and postoperatively. Preoperative assessments included establishing a baseline RDW. Postoperative data collection involved RDW values, arterial blood gases, and radiologic reports within 48 h of surgery to provide comprehensive postoperative data.

### 2.4. Statistical Analysis

Continuous variables are reported as mean ± standard deviation (SD) and compared using the independent Student’s *t*-test. If normality is not assumed (as verified by the Kolmogorov–Smirnov test), medians (interquartile range, IQR) are used, and comparisons are carried out using the Mann–Whitney U test. Categorical variables are expressed as numbers and percentages and compared using the chi-square test or Fisher’s exact test as appropriate. Categorical variables are expressed as numbers and percentages, and Pearson or Spearman correlations are obtained to assess associations. Variables such as operation duration, cardiopulmonary bypass time, aortic clamping time, postoperative RDW levels, and the difference in RDW before and after surgery are included in a univariate analysis to identify the risk factors associated with the occurrence of ALI. Subsequently, variables with *p* < 0.05 in the univariate analysis were then entered into a forward stepwise regression model (Forward: LR) to identify the most parsimonious set of independent predictors. Furthermore, an RCS model was employed to investigate the association between RDW and the risk of CPB-ALI, allowing for the assessment of potential nonlinear relationships between RDW levels and clinical outcomes. Statistical significance is set at *p* < 0.05.

## 3. Results

### 3.1. Demographic and Perioperative Characteristics

A total of 166 pediatric patients undergoing cardiac surgery with cardiopulmonary bypass (CPB) were included in the final analysis. Of these, 55 patients (33.1%) developed postoperative CPB-associated acute lung injury (CPB-ALI) (Figure 1). Demographic characteristics, including age, sex, weight, and surgery types, did not differ significantly between the CPB-ALI and non-ALI (CPB-NALI) groups (*p* > 0.05 for all). However, the CPB-ALI group exhibited significantly prolonged durations of CPB time (mean: 127.76 vs. 96.85 min, *p* < 0.001), aortic cross-clamp time (mean: 78.67 vs. 54.69 min, *p* < 0.001), and total operative time (mean: 5.10 vs. 4.47 h, *p* = 0.003) compared to the CPB-NALI group. Furthermore, CPB-ALI patients required prolonged mechanical ventilation (MV) (median: 19.11 vs. 9.21 h, *p* = 0.014) and longer ICU stays (median: 6.90 vs. 3.98 days, *p* < 0.001), and they exhibited an increased total hospital length of stay (LOS) (median: 21.00 vs. 17.00 days, *p* = 0.001) (Table 1).

### 3.2. Perioperative RDW Dynamics

Preoperative RDW levels did not differ significantly between the CPB-ALI and CPB-NALI groups (mean: 14.28% vs. 13.91%, *p* = 0.076). However, postoperative RDW levels within 48 h were markedly elevated in the CPB-ALI group (15.40% vs. 13.78%, *p* < 0.001). No significant intra-group variation in RDW levels was observed in the CPB-NALI group (13.91% vs. 13.78%, *p* = 0.929), whereas the CPB-ALI group demonstrated a significant postoperative RDW increase (14.28% vs. 15.40%, *p* < 0.001) (Table 2).

### 3.3. RDW as an Independent Predictor of CPB-ALI

Univariate logistic regression analysis identified prolonged operative time (OR: 1.54, 95% CI: 1.17–2.04, *p* = 0.002), CPB duration (OR: 1.02, 95% CI: 1.01–1.02, *p* < 0.001), aortic cross-clamp time (OR: 1.02, 95% CI: 1.01–1.03, *p* < 0.001), postoperative RDW (OR: 1.32, 95% CI: 1.13–1.54, *p* < 0.001), and ΔRDW (OR: 4.51, 95% CI: 2.52–8.06, *p* < 0.001) as significant risk factors for CPB-ALI.

Multivariate analyses confirmed that both postoperative RDW (OR: 1.35, 95% CI: 1.10–1.64, *p* = 0.003) and ΔRDW (OR: 4.22, 95% CI: 2.31–7.72, *p* < 0.001) were independent predictors after adjusting for surgical durations and other perioperative variables (Table 3).

### 3.4. ROC Curve Analysis

**Figure 2:**
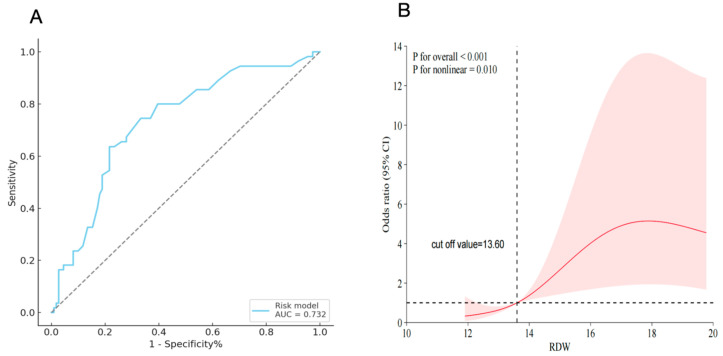
ROC curve and RCS analysis. (**A**) ROC curve analysis for postoperative RDW concentrations and the incidence of CPB-ALI in pediatric patients. solid blue line shows sensitivity versus 1 – specificity; dashed gray line is reference (AUC = 0.732); (**B**) RCS model showing the association between postoperative RDW concentrations and the incidence of CPB-ALI in pediatric patients. Solid red line shows odds ratio across RDW values; shaded area is 95% CI; vertical dashed line indicates cutoff (13.6%). AUC, Area under the curve; CI, confidence interval; RDW, red cell distribution width.

The area under the ROC curve (AUC) for postoperative RDW in predicting CPB-ALI was 0.732 (Figure 2). At a cutoff value of 14.3%, RDW demonstrated optimal sensitivity and specificity. Detailed performance metrics including sensitivity, specificity, positive predictive value (PPV), and negative predictive value (NPV) are presented in Table 4.

### 3.5. Nonlinear Association Between RDW and CPB-ALI Risk

Restricted cubic spline (RCS) analysis revealed a nonlinear dose–response relationship between postoperative RDW and CPB-ALI incidence (*p* for nonlinearity = 0.010; *p* overall < 0.001). The risk of CPB-ALI increased progressively with RDW levels above 13.60% (Figure 2).

### 3.6. Association of RDW with Clinical Outcomes

**Figure 3:**
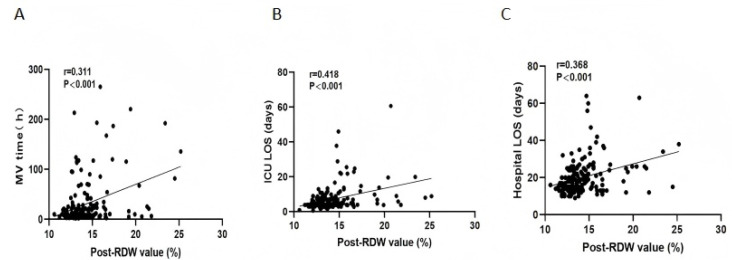
Scatter plots illustrating the association between postoperative RDW concentrations and clinical outcomes. (**A**) Mechanical ventilation (MV) duration, (**B**) intensive-care-unit (ICU) length of stay (LOS), and (**C**) total hospital LOS are presented. RDW, Red cell distribution width; ICU, intensive care unit; LOS, length of stay; MV, mechanical ventilation.

Postoperative RDW levels positively correlated with the duration of mechanical ventilation (*r* = 0.311, *p* < 0.001), ICU length of stay (*r* = 0.418, *p* < 0.001), and hospital LOS (*r* = 0.368, *p* < 0.001) (Figure 3).

## 4. Discussion

This study demonstrates that the elevated postoperative red cell distribution width (RDW) is significantly associated with the development of cardiopulmonary bypass-associated acute lung injury (CPB-ALI) in pediatric patients undergoing cardiac surgery. The multivariate logistic regression model identified postoperative RDW (OR: 1.35, 95% CI: 1.10–1.64, *p* = 0.003) and ΔRDW (OR: 4.22, 95% CI: 2.31–7.72, *p* < 0.001) as independent predictors of CPB-ALI, even after adjusting for surgical duration and other perioperative variables. These findings align with emerging evidence implicating RDW as a prognostic marker in systemic inflammatory and oxidative stress-related pathologies, such as acute respiratory distress syndrome (ARDS) and acute pancreatitis-associated lung injury [25,26,27]. Notably, our study expands on the existing literature by focusing on the pediatric population and introducing a nonlinear analysis of RDW, demonstrating that the risk of CPB-ALI increases disproportionately when RDW exceeds 13.60%.

The association between RDW and CPB-ALI may be driven by pathophysiological processes inherent to cardiopulmonary bypass. CPB induces systemic inflammation, oxidative stress, and erythrocyte membrane destabilization, which collectively impair red blood cell (RBC) deformability and promote heterogeneous RBC size distribution [29,30,31]. An elevated RDW may thus reflect the cumulative burden of these insults, serving as a surrogate marker for subclinical inflammation and tissue hypoxia. This hypothesis is supported by studies linking RDW to endothelial dysfunction and cytokine release in critical illness [20,25,29]. However, the absence of direct measurements of inflammatory mediators (e.g., IL-6, TNF-*α*) in this study limits mechanistic conclusions.

The predictive performance of the postoperative RDW (AUC: 0.732) suggests its potential role as an adjunctive biomarker for the early identification of high-risk pediatric patients. While its standalone predictive capacity is moderate, integrating RDW with established clinical parameters (e.g., CPB duration, PaO_2_/FiO_2_ ratio) could enhance risk stratification and guide personalized interventions, such as targeted anti-inflammatory therapies or optimized ventilator strategies. The correlation between elevated RDW and prolonged mechanical ventilation (*r* = 0.311, *p* < 0.001), ICU stay (*r* = 0.418, *p* < 0.001), and hospital length of stay (*r* = 0.368, *p* < 0.001) further underscores its prognostic relevance in resource allocation and postoperative care planning.

This study has several limitations. First, its retrospective single-center design introduces potential selection bias and limits generalizability. Second, the exclusion of patients with preoperative lung injury or comorbidities may underestimate RDW’s predictive value in broader populations. Third, the lack of serial RDW measurements precludes an analysis of temporal trends in RDW dynamics. Future prospective multicenter studies should validate these findings in diverse cohorts, incorporate longitudinal biomarker profiling (e.g., RDW, inflammatory cytokines like IL-6 or TNF-*α*), and explore combinatorial models integrating RDW with imaging or genomic data. Mechanistic investigations, including in vitro models of CPB-induced erythrocyte injury, are also warranted to elucidate causal pathways linking RDW to pulmonary dysfunction.

## 5. Conclusions

In conclusion, postoperative RDW elevation serves as an independent predictor of CPB-ALI and adverse clinical outcomes in pediatric cardiac surgery patients. Its accessibility, cost-effectiveness, and correlation with systemic inflammation position RDW as a promising tool for early risk assessment. However, its integration into clinical practice requires validation through rigorous prospective studies and the development of multimodal prediction algorithms.

## Figures and Tables

**Table 1:** Demographic and clinical characteristics of the patients enrolled in the study cohort.

Characteristics	CPB—NALI (n = 111, 66.9%)	CPB—ALI (n = 55, 33.1%)	*p* Value
Age (days)	1132.00 (415.00–2099.00)	624.00 (376.00–1696.00)	*p* = 0.091
Weight (kg)	12.00 (9.00–18.00)	10.00 (9.00–15.00)	*p* = 0.054
Gender, male	60 (54.1%)	33 (60.0%)	*p* = 0.47
Hospital LOS (d)	17.00 (13.00–22.00)	21.00 (15.00–27.00)	*p* = 0.001
ICU LOS (d)	3.98 (3.66–6.58)	6.90 (4.00–11.72)	*p* < 0.001
Operative time (h)	4.47 ± 1.12	5.10 ± 1.30	*p* = 0.003
CPB time (min)	96.85 ± 38.13	127.76 ± 52.27	*p* < 0.001
Aortic clamping time (min)	54.69 ± 33.50	78.67 ± 39.95	*p* < 0.001
MV time (h)	9.21 (5.57–24.28)	19.11 (7.29–47.95)	*p* = 0.014
ASD, VSD repair surgery (%)	48 (43.2%)	17 (30.9%)	*p* = 0.133
Vascular surgery (%)	31 (27.9%)	11 (20.0%)	*p* = 0.344
Tetralogy of Fallot repair surgery (%)	13 (11.7%)	12 (21.8%)	*p* = 0.107
Valve repair surgery (%)	10 (9.0%)	3 (5.5%)	*p* = 0.548
Outflow tract surgery (%)	4 (3.6%)	6 (10.9%)	*p* = 0.084
Other surgery (%)	5 (4.5%)	6 (10.9%)	*p* = 0.182

Data are presented as median (interquartile range) or mean ± standard deviation for continuous variables and as number (percentage) for categorical variables. ASD, Atrial septal defect; CPB, cardiopulmonary bypass; LOS, length of stay; MV, mechanical ventilation; VSD, ventricular septal defect.

**Table 2:** Comparison of the mean levels of pre- and postoperative RDW concentrations.

Testing Period	Control	Study	Difference (Control–Study)	*p* Value (Control vs. Study)
Preoperative	13.91 ± 2.60	14.28 ± 2.30	−0.37±0.41 (−1.18, 0.45)	0.076
Postoperative	13.78 ± 2.15	15.40 ± 2.72	−1.62±0.42 (−2.45, −0.78)	<0.001
Difference (pre–post)	0.13±1.14 (−0.08, 0.35)	−1.12±1.36 (−1.49, −0.75)	–	–
*p* value (pre vs. post)	0.929	<0.001	–	–

Data are expressed as mean ± standard deviation or mean difference with 95% confidence intervals in parentheses. RDW, red blood cell distribution width.

**Table 3:** Univariate and multivariate logistic regression analyses for risk factors associated with CPB-ALI.

Characteristics	Univariate		Multivariate	
	OR (95% CI)	*p*-Value	OR (95% CI)	*p*-Value
CPB time (min)	1.02 (1.01–1.02)	<0.001	1.02 (1.01–1.03)	0.001
Clamp time (min)	1.02 (1.01–1.03)	<0.001	–	0.643
Operative time (min)	1.54 (1.17–2.04)	0.002	–	0.896
Post-RDW value (%)	1.32 (1.13–1.54)	<0.001	1.35 (1.10–1.64)	0.003
Pre–post-RDW change (%)	4.51 (2.52–8.06)	<0.001	4.22 (2.31–7.72)	<0.001

Odds ratios (ORs) and 95% confidence intervals (CIs) were calculated using univariate and multivariate logistic regression analyses. Variables with p<0.05 in univariate analysis were entered into the multivariate model. p<0.05 was considered statistically significant. CPB, Cardiopulmonary bypass; RDW, red blood cell distribution width.

**Table 4:** Performance of postoperative RDW levels for the diagnosis of CPB-ALI.

	Area Under ROC Curve (95% CI)	At a Cutoff Value of 14.3%			
		Sensitivity (95% CI)	Specificity (95% CI)	PPV (95% CI)	NPV (95% CI)
Postoperative RDW levels	0.732 (0.658–0.797)	0.636 (0.605–0.668)	0.784 (0.745–0.823)	0.593 (0.564–0.623)	0.813 (0.773–0.854)

The diagnostic performance of postoperative RDW levels for predicting CPB-ALI was evaluated using receiver operating characteristic (ROC) curve analysis. AUC, Area under the curve; RDW, red blood cell distribution width; CPB-ALI, cardiopulmonary bypass-associated acute lung injury.

## Data Availability

The data presented in this study are available upon request from the corresponding author. The data are not publicly available due to ethical and privacy restrictions.

## Abbreviations

The following abbreviations are used in this manuscript:

ASD	Atrial septal defect;
VSD	Ventricular septal defect;
CPB	Cardiopulmonary bypass;
ALI	Acute lung injury;
CPB-ALI	Cardiopulmonary bypass-associated acute lung injury;
CPB-NALI	Cardiopulmonary bypass-associated non-acute lung injury;
RDW	Red cell distribution width;
ICU	Intensive care unit;
LOS	Length of stay;
MV	Mechanical ventilation;
AUC	Area under the curve;
RCS	Restricted cubic spline;
ROC	Receiver operating characteristic;
OR	Odds ratio;
CI	Confidence interval.
